# Assessing the modification impact of vaccination on the relationship of the Discomfort Index with hand, foot, and mouth disease in Guizhou: A multicounty study

**DOI:** 10.1371/journal.pntd.0012008

**Published:** 2024-07-01

**Authors:** Jie Sun, Wangjian Zhang, Guanghai Yao, Jing Gu, Wenjing Wu, Dan Wang, Zhicheng Du, Yuantao Hao

**Affiliations:** 1 Department of Medical Statistics, School of Public Health & Center for Health Information Research & Sun Yat-sen Global Health Institute, Sun Yat-sen University, Guangzhou, China; 2 Institute for the Control of Infectious Diseases, Guizhou Center for Disease Control and Prevention, Guiyang, Guizhou, China; 3 Guangzhou Joint Research Center for Disease Surveillance and Risk Assessment, Sun Yat-sen University & Guangzhou Center for Disease Control and Prevention, Guangzhou, China; 4 Peking University Center for Public Health and Epidemic Preparedness & Response, Peking University, Beijing, China; 5 Department of Epidemiology & Biostatistics, School of Public Health, Peking University, Beijing, China; 6 Key Laboratory of Epidemiology of Major Diseases (Peking University), Ministry of Education, Peking University, Beijing, China; University of Calgary, CANADA

## Abstract

**Background:**

Hand, foot, and mouth disease (HFMD) is a major public health issue in China while temperature and humidity are well-documented predictors. However, evidence on the combined effect of temperature and humidity is still limited. It also remains unclear whether such an effect could be modified by the enterovirus 71 (EV71) vaccination.

**Methods:**

Based on 320,042 reported HFMD cases during the summer months between 2012 and 2019, we conducted a study utilizing Distributed Lag Non-Linear Models (DLNM) and time-varying DLNM to examine how China’s HFMD EV71 vaccine strategy would affect the correlation between meteorological conditions and HFMD risk.

**Results:**

The incidence of HFMD changed with the Discomfort Index in an arm-shaped form. The 14-day cumulative risk of HFMD exhibited a statistically significant increase during the period of 2017–2019 (following the implementation of the EV71 vaccine policy) compared to 2012–2016 (prior to the vaccine implementation). For the total population, the range of relative risk (*RR*) values for HFMD at the 75th, 90th, and 99th percentiles increased from 1.082–1.303 in 2012–2016 to 1.836–2.022 in 2017–2019. In the stratified analyses, Han Chinese areas show stronger relative growth, with *RR* values at the 75th, 90th, and 99th percentiles increased by 14.3%, 39.1%, and 134.4% post-vaccination, compared to increases of 22.7%, 41.6%, and 38.8% in minority areas. Similarly, boys showed greater increases (24.4%, 47.7%, 121.5%) compared to girls (8.1%, 28.1%, 58.3%). Additionally, the central Guizhou urban agglomeration displayed a tendency for stronger relative growth compared to other counties.

**Conclusions:**

Although the EV71 vaccine policy has been implemented, it hasn’t effectively controlled the overall risk of HFMD. There’s been a shift in the main viral subtypes, potentially altering population susceptibility and influencing HFMD occurrences. The modulating effects of vaccine intervention may also be influenced by factors such as race, sex, and economic level.

## Author summary

Hand, foot, and mouth disease (HFMD) is one of the most common childhood enteric infections worldwide and an important public health problem in China. In this study, we analyzed the relationships among HFMD cases, meteorological factors, and vaccine factors using time-varying distributed lag nonlinear models. We found that the susceptibility of the population may have changed after the implementation of the HFMD EV71 vaccine policy, altering the relationship between HFMD and meteorological factors. The modifying effects of vaccine interventions may be influenced by variables such as race, sex, and economic level. This provides valuable information for the prevention of the disease and policy decisions.

## Introduction

Hand, foot, and mouth disease (HFMD) is a significant public health concern [1], particularly in Asian countries, including China [2,3]. Since 2008, it has been categorized as a class C legal infectious disease in China, with the highest incidence rate among all diseases [4]. The annual reported incidence rate has shown an increasing trend, and severe cases have also been reported [5].

Meteorological factors play a crucial role in the occurrence and prevalence of infectious diseases, including HFMD. Temperature and relative humidity have been identified as the primary influencing factors for HFMD [6,7]. Previous studies have mainly focused on the single effects of temperature or relative humidity, while the joint effect of these factors has received less attention. Recently, composite indices, such as Humidex [8] and THIa [9], have been developed to estimate the combined effect of temperature and relative humidity on HFMD. However, research utilizing similar composite indices in this area remains relatively scarce.

In addition to meteorological factors, the implementation of the Enterovirus 71 (EV71) vaccination initiative, which began in late 2016, has had an impact on the prevention of HFMD in China [10]. The vaccine is effective against EV71 infection and may also influence human susceptibility to other enteroviruses associated with HFMD [11,12]. Although previous studies have highlighted the important role of EV71 vaccines in reducing EV71-associated HFMD, there remains a lack of comprehensive evaluation of their effectiveness in a broader population. Currently, there is limited evidence on how the implementation of the EV71 vaccine may modify the relationship between meteorological factors and HFMD. Although the importance of the EV71 vaccine is well recognized, a more comprehensive evaluation is needed.

Furthermore, there is a paucity of pertinent research conducted in the underdeveloped western region of China, with the majority of previous studies concentrating on economically developed cities in the eastern and southern regions of the country. To address these critical gaps, we conducted a county-level time series analysis of HFMD in Guizhou province. Our study aimed to examine the relationship between the Discomfort Index (a combined measure of temperature and relative humidity) and the incidence of HFMD. Additionally, we evaluated the potential influence of EV71 vaccine implementation on this relationship. We also identified potential moderating effects of residential, sex, and economic factors. Our findings provide valuable insights for assessing vaccine efficacy and developing targeted vaccination strategies. This study fills a critical knowledge gap regarding the comprehensive impact of meteorological factors on HFMD and the modifying role of EV71 vaccine policy, thereby making a significant contribution to the existing literature.

## Methods

### Setting

Guizhou Province, situated in southwestern China (latitude 24°37’~29°13’N, longitude 103°36’~109°35’E), is characterized by a lower level of urbanization and a significant population of ethnic minorities, making it a region known for its ethnic diversity. According to the Köppen climate classification, Guizhou Province can be predominantly categorized into two climate types: Stheubtropical Humid Climate (Cfa) in the southeastern part and Subtropical Monsoon Climate (Cwa) in the southwestern part [13]. It consists of 88 counties, covering an area of 176,167 square kilometers, and has a resident population of approximately 3,856,210,000. According to the 7th National Census, the Han Chinese population makes up 63.56% of the resident population, while the remaining 36.44% comprises various ethnic minorities.

### Data sources

#### HFMD data

We collected HFMD data in Guizhou Province from the Chinese Disease Prevention and Control Information System. Our study specifically targeted children aged 0–15 years from 2012 to 2019, with a particular emphasis on the summer months spanning from May to September. Each case encompassed essential details such as sex, age, current address code, and onset time. We compiled the daily count of cases per county based on the onset date, using address information from 88 counties in the province.

#### Meteorological data

Meteorological data for the study period were collected from the China Meteorological Data Sharing Service (http://data.cma.cn/site/index.html). We obtained daily records of average temperature, relative humidity, air pressure, and precipitation from 34 national surface weather stations in Guizhou province. The weather stations were matched to the 88 counties based on latitude and longitude. In cases where multiple weather stations were available, we selected the station closest to the regional center. For counties without dedicated weather stations, we utilized data from the nearest available station. To handle missing values, we employed a method of filling them with the average data from the two preceding and following days.

#### The Discomfort Index definition

Based on the meteorological variables available to us, this study focuses on the combined effects of temperature and relative humidity. The Discomfort Index is a composite index that quantifies an individual’s thermal discomfort due to temperature and relative humidity [14]. The equation for calculation is as follows:

Discomfortindex=T−(0.55−0.0055×RH)(T−14.5) (1)

where *T* stands for temperature and *RH* stands for relative humidity. This index is considered to represent human discomfort in a hot environment [14]. The higher the Discomfort Index, the more pronounced the negative impact of hot and humid environmental conditions on human comfort, although the acclimation of the population may vary across regions, sexes, ethnicities, etc.

#### Vaccine implementation

In 2010, the inactivated EV71 vaccine was successfully developed and introduced to the market in 2016 [15]. Following this, in 2017, the EV71 vaccination was initiated in Guizhou Province, focusing on children aged between 6 months and 5 years [5]. Notably, this vaccination effort is self-funded and voluntary [16]. The date January 1, 2017, serves as the cutoff point for both the period preceding and following the implementation of the vaccine [15,16].

### Statistical analysis

We employed Distributed Lag Non-Linear Models (DLNM) and time-varying DLNM for our analysis [17,18]. The specific analytical steps were as follows:

#### First-stage analysis

We applied quasi-Poisson generalized additive regression (GAM) combined with DLNM to fit the relationship of the Discomfort Index with HFMD [19]. We defined specific model for the Discomfort Index exposure as follows:

$$\begin{array}{c} Y_{t}=Poisson\;(u_{t})\\ \mathrm{Log}[E(Y_{t})]=a+cb(theDicomfortIndex)+ns(airpressure,df=3)+ns(precipitation,df=3)\\ +ns(doy,df=4):factor(year)+dow+holiday \end{array}$$ (2)

Where *Y_t_* represents the daily count of HFMD cases on day *t*. The Discomfort Index is the exposure factor in our study. The bi-dimensional exposure-lag-response relationship between the Discomfort Index and HFMD is characterized using a cross-basis function (*cb*) [19,20]. The cross-basis consists of a combination of an exposure-response function and a lag-response function, with 4 degrees of freedom (*df*) each [17]. Following prior research and the longest incubation period of HFMD, we set the lag effect of the Discomfort Index to 0–14 days [8,20]. For the two covariates, air pressure and precipitation, we modeled with 3 *df* natural cubic splines [20]. Additionally, 4 *df* are used to capture the relationship with the date (day of year, *doy*) [20]. Moreover, we adjusted for the effect of day of week (*dow*), treating it as a categorical variable. Furthermore, the holiday indicator (*holiday*, including school holidays and public holidays) is a binary variable used to control for public holidays [19,20].

We further employed a time-varying DLNM to investigate the time-varying trends in the relationship between the Discomfort Index exposure and HFMD before and after the implementation of the EV71 vaccination policy [17]. Based on the implementation timing of the EV71 vaccine policy, we divided the entire study period into two parts (2012–2016 and 2017–2019). To assess the effect modification of the EV71 vaccination, we introduced time-exposure interaction terms into the model, selecting the central days of the corresponding summer months (July 16th in 2014 and 2018) as reference points. We then utilized linear interaction terms between time and cross-basic exposure variables to reveal time-varying trends, reflecting the effect modification of vaccine implementation policy. Furthermore, we subjected this interaction term to a multivariate Wald test to test changes in the time trend of the cumulative Discomfort Index exposure and HFMD. And this time variation reflects changes in vaccine implementation policies. The minimum incidence percentile represented the average association over the entire study period, estimating the minimum value of the exposure-response curve. We calculated the relative risks (*RR*s) of the 75th, 90th, and 99th percentiles compared to the minimum incidence percentile. Additionally, we conducted stratified analyses based on the residential, sex, and economic zone characteristics of the study population.

#### Second-stage analysis

Subsequently, we employed multivariate meta-regression to pool the county-specific level estimates obtained from the first-stage analysis [21]. Our analysis primarily focused on the cumulative exposure-response relationship while also incorporating the modifying effect of vaccine implementation policy.

#### Sensitivity analysis

To ensure the reliability of the study results, we conducted sensitivity analyses, exploring *df* values ranging from 4 to 6 for both the exposure-response function and the lag-response function. Simultaneously, we investigated covariates, such as air pressure and precipitation, with *df* values varying from 3 to 5. Subsequently, we selected the optimal parameters using the quasi-Poisson model’s Akaike Information Criterion (Q-AIC), ensuring the rigor of the selection process. Additionally, we computed Humidex, another commonly used indicator reflecting the combined effect of temperature and relative humidity, based on the formula provided in previous studies [22]. We evaluated the relationship between Humidex and HFMD, comparing it with that for the Discomfort Index. Furthermore, we strengthened the analysis specifically for individuals under 5 years of age (S3 and S4 Tables).

For the statistical analyses, R software (version 4.2.1) was used. Specifically, the *data*.*table*, *rSPARCS*, *dlnm*, *mvmeta*, and *ThermIndex* packages were used for data manipulation, while the *ggplot2* packages was used for result visualization. A significance level of *P* < 0.05 was considered statistically significant for two-sided tests.

## Results

### Descriptive statistic

Table 1 presents the total of 320,042 reported HFMD cases in Guizhou Province during the summer months from 2012 to 2019. The trend analysis of HFMD incidence from 2012 to 2019 is presented in S1 Fig. Despite the implementation of vaccination in 2017, the figure illustrates that the incidence of HFMD continues to increase. Specifically, there were 172,119 reported cases from 2012 to 2016, while 147,923 cases were reported from 2017 to 2019. During the period from 2012 to 2019, 256,411 cases (80.1%) occurred in 42 county-level Han Chinese areas, while 63,631 cases (19.9%) were reported in 46 county-level minority’s areas. Girls accounted for 127,941 cases (40.0%), and boys accounted for 192,101 cases (60.0%). Within Guizhou Province, 33 counties in the urban agglomeration in central Guizhou subgroup reported 220,138 cases (68.8%), while the remaining 99,904 cases (31.2%) were reported in 55 counties outside this urban agglomeration. Overall, the distribution of the Discomfort Index ranged from 12.563 to 26.264. Between 2012 and 2016, the Discomfort Index ranged from 12.798 to 26.211, with a median of 21.909. Meanwhile, between 2017 and 2019, the Discomfort Index ranged from 13.208 to 25.994, with a median of 22.390. Furthermore, an analysis of the composition of HFMD pathogens from 2012 to 2019 revealed a decline in the proportion of EV71 viruses and an increasing proportion of coxsackievirus A16 and other enteric types (S5 Fig).

**Table 1:** Descriptive statistics of HFMD cases in Guizhou Province, China, during the summer months of 2012–2019.

Stratification	Counties(n)	Total cases (n)	Period (year)	The Discomfort Index distribution				
				Minimum	25th	Median	75th	Maximum
**Total**	88	320042	2012–2019	12.563	20.011	22.085	23.638	26.264
		172119	2012–2016	12.798	19.953	21.909	23.567	26.211
		147923	2017–2019	13.208	20.143	22.390	23.727	25.994
**Residential area**								
** Han Chinese areas**	42	256411	2012–2019	12.078	19.300	21.449	23.160	25.853
			2012–2016	12.426	19.245	21.270	23.091	25.804
			2017–2019	12.392	19.434	21.763	23.248	25.563
** Minority areas**	46	63631	2012–2019	13.006	20.661	22.667	24.075	26.639
			2012–2016	13.137	20.600	22.493	24.003	26.583
			2017–2019	13.953	20.791	22.962	24.164	26.388
**Sex**								
** Girl**	88	127941	2012–2019	12.563	20.011	22.085	23.638	26.264
			2012–2016	12.798	19.953	21.909	23.567	26.211
			2017–2019	13.208	20.143	22.390	23.727	25.994
** Boy**	88	192101	2012–2019	12.563	20.011	22.085	23.638	26.264
			2012–2016	12.798	19.953	21.909	23.567	26.211
			2017–2019	13.208	20.143	22.390	23.727	25.994
**Economic zone**								
** Urban agglomeration in central Guizhou**	33	220138	2012–2019	12.161	19.334	21.433	23.015	25.692
			2012–2016	12.317	19.236	21.238	22.950	25.676
			2017–2019	12.467	19.514	21.751	23.104	25.407
** Other counties**	55	99904	2012–2019	12.804	20.418	22.477	24.012	26.607
			2012–2016	13.087	20.384	22.312	23.938	26.532
			2017–2019	13.653	20.521	22.773	24.100	26.347

In sensitivity analyses, we found that the exposure-response relationship between Humidex and HFMD was similar to that between the Discomfort Index and HFMD (S4 Fig). Additionally, our study specifically focused on the 0–5-year-old age group, which included a total of 240,863 cases of HFMD. The descriptive statistics for this group can be found in S3 and S4 Tables.

### Time-constant effects estimate

**Fig 1:**
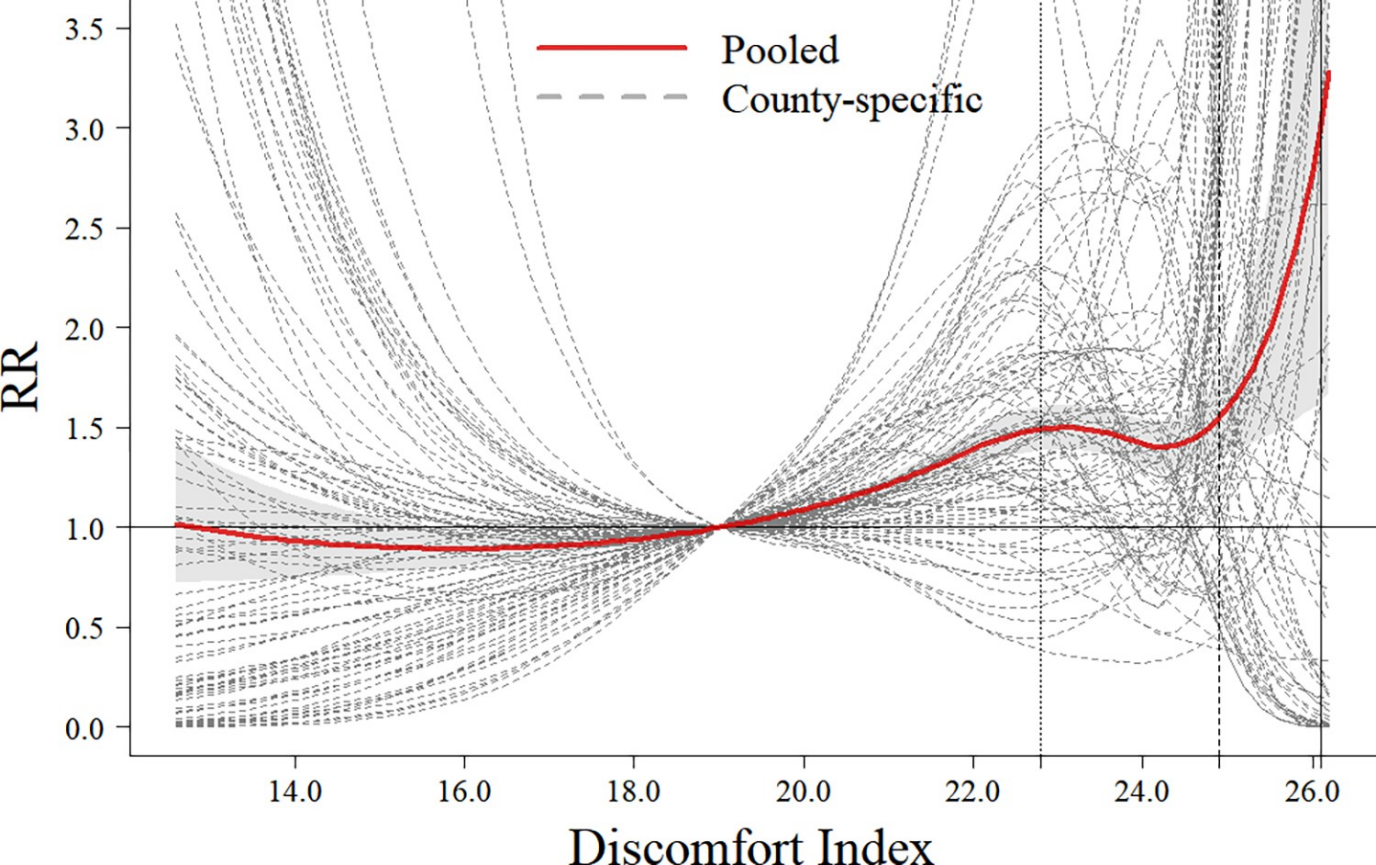
The pooled and county-specific cumulative risk curves of the relationships between the Discomfort Index and HFMD in Guizhou Province, China, 2012–2019. Note: 1) The different types of vertical lines represent the 75th, 90th, and 99th percentiles of the Discomfort Index distribution, respectively. 2) The gray area represents the 95% CI, with a reference point set at the 50th percentile of the Discomfort Index, which is 19.4.

The results presented a nonlinear relationship between the Discomfort Index and HFMD, characterized by an arm-shaped pattern (Fig 1). The overall pooled estimates suggest a slight decrease in risk at the Discomfort Index of approximately 24, followed by a notable increase in risk at exceptionally high levels. Specifically, the 75th, 90th, and 99th percentiles of the Discomfort Index were 22.8, 24.9, and 26.1, respectively. The corresponding *RR* values were 1.491 (95% confidence interval (CI): 1.385, 1.605), 1.550 (95% Cl: 1.357, 1.769), and 3.014 (95% Cl: 1.633, 5.564), respectively.

### Time-varying effects estimate

Table 2 presents the minimum incidence percentile for the boys’ subgroup is the 11th percentile, while for the total population and other subgroups, the minimum incidence percentile is the 10th percentile. Notably, statistically significant temporal trends were observed in the total population for both the periods of 2012–2016 and 2017–2019 (*P* <0.001 for time interaction, Wald test). Specifically, the point estimates of *RR* for HFMD increased from 1.303, 1.183, and 1.082 in the period 2012–2016 to 1.836, 1.906, and 2.022 in the period 2017–2019 at the 75th, 90th, and 99th percentiles, respectively.

**Table 2:** Temporal changes of the *RR* of HFMD in the total and subgroups population in different periods (total, 2012–2016, and 2017–2019).

Stratification	The minimum incidence percentile	Period (year)	75th *RR*	90th *RR*	99th *RR*	*P-*Value^a^
**Total**	10th	2012–2019	1.673 (1.512, 1.851)	1.705 (1.511, 1.923)	1.715 (1.478, 1.990)	
		2012–2016	1.303 (1.099, 1.544)	1.183 (0.985, 1.422)	1.082 (0.872, 1.342)	<0.001
		2017–2019	1.836 (1.628, 2.069)	1.906 (1.662, 2.185)	2.022 (1.720, 2.376)	
**Residential area**						
** Han Chinese areas**	10th	2012–2016	1.465 (1.330, 1.613)	1.196 (1.084, 1.320)	1.048 (0.897, 1.223)	<0.001
		2017–2019	1.675 (1.502, 1.869)	1.664 (1.487, 1.862)	2.457 (1.949, 3.098)	
** Minority areas**	10th	2012–2016	1.517 (1.155, 1.993)	1.343 (0.983, 1.837)	1.553 (0.970, 2.485)	0.021
		2017–2019	1.861 (1.591, 2.178)	1.902 (1.613, 2.244)	2.155 (1.642, 2.827)	
**Sex**						
** Girl**	10th	2012–2016	1.575 (1.334, 1.859)	1.318 (1.066, 1.630)	1.135 (0.868, 1.485)	<0.001
		2017–2019	1.703 (1.521, 1.907)	1.689 (1.496, 1.907)	1.797 (1.376, 2.347)	
** Boy**	11th	2012–2016	1.475 (1.284, 1.695)	1.239 (1.051, 1.461)	1.128 (0.894, 1.424)	<0.001
		2017–2019	1.835 (1.647, 2.045)	1.830 (1.642, 2.040)	2.499 (2.054, 3.041)	
**Economic zone**						
** Urban agglomeration in central Guizhou**	10th	2012–2016	1.567 (1.354,1.812)	1.329 (1.109,1.593)	1.161 (0.901,1.497)	<0.001
		2017–2019	1.565 (1.377,1.778)	1.527 (1.336,1.745)	2.285 (1.853,2.818)	
** Other counties**	10th	2012–2016	1.360 (1.070,1.729)	1.123 (0.838,1.505)	1.216 (0.866,1.708)	<0.001
		2017–2019	1.931 (1.704,2.188)	1.982 (1.734,2.267)	2.181 (1.659,2.868)	

^a^: A multivariate Wald test with interaction terms was used to assess the significance of changes before and after the implementation of the EV71 vaccine policy. The null hypothesis is that there is no difference in *RR* before and after the implementation of the EV71 vaccine policy.

In addition, stratified analysis based on the residential area, sex, and economic zone characteristics of the study population revealed that, compared to the period 2012–2016, all subgroups demonstrated a significant increase in *RR* values during the period 2017–2019 (*P*<0.05).

**Fig 2:**
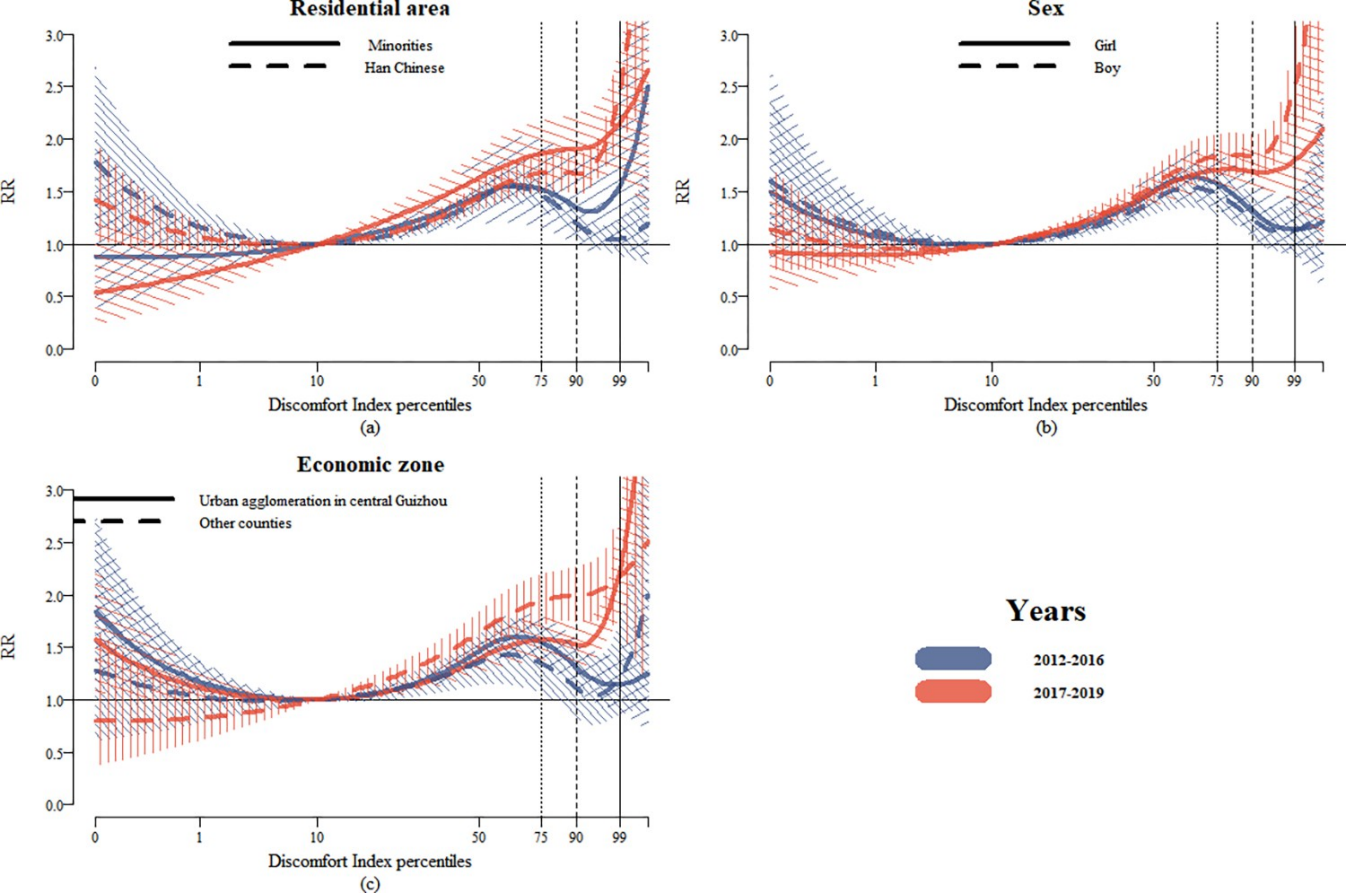
Cumulative risk curves of the Discomfort Index and HFMD relationship for 2012–2016 and 2017–2019 in subgroups (with 95% Cl). (a) Residential area stratification. (b) Sex stratification. (c) Economic zone stratification. Note: 1) The different types of vertical lines represent the 75th, 90th, and 99th percentiles of the Discomfort Index distribution, respectively. 2) The cumulative risk curves obtained from the time-varying DLNM are compared between the time periods 2012–2016 (blue) and 2017–2019 (red).

Stratified by area of residence, *RR* values for the 75th, 90th, and 99th percentiles of the Discomfort Index in Han Chinese areas during the period 2017–2019 were 1.675 (95% Cl: 1.502, 1.869), 1.664 (95% Cl: 1.487, 1.862), and 2.457 (95% Cl: 1.949, 3.098). These values were higher than the corresponding *RR* values for the period 2012–2016, which were 1.465 (95% Cl: 1.330, 1.613), 1.196 (1.084, 1.320), and 1.048 (95% Cl: 0.897, 1.223), respectively. A similar trend was observed in minority areas. *RR* values for the 75th, 90th, and 99th percentiles in minority areas during the period 2017–2019 were 1.861 (95% Cl: 1.591, 2.178), 1.902 (95% Cl: 1.613, 2.244), and 2.155 (95% Cl: 1.642, 2.827). These values were higher than the corresponding *RR* values for the period 2012–2016, which were 1.517 (95% Cl: 1.155, 1.993), 1.343 (95% Cl: 0.983, 1.837), and 1.553 (95% Cl: 0.970, 2.485), respectively. Furthermore, the *RR* values for the 75th, 90th, and 99th percentiles in minority areas during the period 2017–2019 were higher than the corresponding *RR* values in Han Chinese areas, which are presented in Table 2. This trend is also evident in Fig 2A. However, Han Chinese areas exhibited a more pronounced relative increase in *RR* values. Specifically, after the implementation of the vaccine policy (2017–2019), the 75th, 90th, and 99th percentile *RR* values showed increments of 14.3%, 39.1%, and 134.4% in Han Chinese areas, while minority areas showed increases of 22.7%, 41.6%, and 38.8%, respectively.

The sex-stratified results indicated that boys and girls had similar *RR* values during the period of 2012–2016 (Table 2 and Fig 2B). However, from the period 2012–2016 to 2017–2019, boys experienced a higher increase in *RR* values compared to girls. Specifically, the increases in *RR* values for the 75th, 90th, and 99th percentiles were 24.4%, 47.7%, and 121.5%, respectively, for boys, compared to 8.1%, 28.1%, and 58.3% for girls. It’s worth noting that the *RR* values for the 75th, 90th, and 99th percentiles in boys during the period 2017–2019 were 1.835 (95% Cl: 1.647, 2.045), 1.830 (95% Cl: 1.642, 2.040), and 2.499 (95% Cl: 2.054, 3.041). These values were higher than the corresponding *RR* values for the period 2012–2016, which were 1.475 (95% Cl: 1.284, 1.695), 1.239 (95% Cl: 1.051, 1.461), and 1.128 (95% Cl: 0.894, 1.424), respectively. A similar trend was observed for the girl subgroup.

Between the periods of 2012–2016 and 2017–2019, there was a greater increase in *RR* values for the other counties subgroup compared to the urban agglomeration in central Guizhou subgroup. The *RR* values for the 75th, 90th, and 99th percentiles in the other counties subgroup increased by 42.0%, 76.5%, and 79.4%, respectively. In contrast, the changes in *RR* values for the same percentiles in the urban agglomeration in central Guizhou subgroup were -0.1%, 14.9%, and 96.8%, respectively. Notably, the *RR* values for the Discomfort Index percentiles ranging from the 10th to the 98th percentile were higher in the other counties subgroup compared to the urban agglomeration in central Guizhou subgroup. However, under the extreme Discomfort Index conditions (98th–100th percentile), the urban agglomeration in central Guizhou exhibited a more pronounced increase (Fig 2C). Specifically, the urban agglomeration in central Guizhou subgroup had a higher *RR* value of 2.285 (95% CI: 1.853, 2.818) at the 99th percentile during the 2017–2019 period, while the corresponding *RR* for the other counties subgroup was 2.181 (95% CI: 1.659, 2.868).

We observed a nonlinear relationship in the risk curves for subpopulations (Fig 2). Moreover, we observed that at the extreme Discomfort Index (99th percentile), the *RR* values were significantly higher across all subgroups in both two periods. There was a particularly notable increase during 2017–2019. Within both the total population and subgroups, exposure to the Discomfort Index was found to be associated with a lagged period of 0–14 days, which contributed to the risk of HFMD (S2 and S3 Figs).

The major conclusions of this study are reliable. We determined the *df* values for the exposure-response function and the lag-response function through sensitivity analysis (S1 Table). Additionally, we also conducted sensitivity analyses to select the *df* values for the covariates of air pressure and precipitation (S2 Table). Furthermore, we observed that the results for the under-5 population were similar to those reported in the current study (S3 and S4 Tables).

## Discussion

Quantifying risk factors and evaluating the impact of vaccine interventions are essential components of research on HFMD and child health. Our study sheds light on the detrimental impact of the Discomfort Index, a composite meteorological variable, on HFMD incidence. Furthermore, we demonstrate that the implementation of the EV71 vaccine policy modifies the relationship between the Discomfort Index and HFMD incidence analysis. The findings from our study provide comprehensive and interpretable evidence concerning the intricate connections between vaccine policy, meteorological factors, and HFMD risk. Moreover, our study incorporates multiple stratified analyses, offering comprehensive evidence on the intricate connections between vaccine policy, meteorological factors, and HFMD risk, and deepening our understanding of this domain.

Past studies have found that the number of cases of HFMD increases significantly with increasing temperature and relative humidity [23,24]. This association can be explained by underlying pathological mechanisms involving pathogens, hosts, and environmental factors [25]. Hosts become infected with HFMD by direct contact with respiratory droplets, feces, or contaminated environments containing the virus [26]. As the persistence of viruses in the environment extends, there is an increased probability of their transmission. Nevertheless, the occurrence of these transmissions is dependent on a variety of environmental variables, including relative humidity and temperature [27]. Warmer climates and increased humidity have been found to substantially enhance the viability of enteroviruses [24]. In addition, ambient temperature affects host behavior, with adolescents being more active during warmer months, thereby increasing their exposure to pathogens [27,28]. Our study provides evidence for the combined effects of temperature and relative humidity on HFMD. We emphasize the nonlinear relationship between the Discomfort Index and the risk of HFMD, which is consistent with previous findings [8,9]. Using composite indices like the Discomfort Index, we confirmed that the risk of HFMD increased significantly with increasing temperature and relative humidity [29]. Based on our results, we propose two recommendations. Firstly, close monitoring of weather conditions and timely measures, such as adjusting indoor temperature and humidity, are essential for mitigating HFMD susceptibility among children. Secondly, educating and raising awareness among children about the potential health risks associated with extreme weather factors are imperative, fostering self-protective awareness and behaviors.

The risk of HFMD has significantly increased even after the implementation of the EV71 vaccine policy, indicating that the execution of the EV71 vaccine policy has not effectively mitigated the risk. Several factors may contribute to this outcome. Firstly, the primary virus causing HFMD has undergone changes, with a decrease in cases related to the EV71 virus and an increase in cases caused by other enteroviruses (EV) [20]. This trend has consistently been observed in the pathogen distribution across different years in Guizhou province. Such a changing pattern suggests that the EV71 vaccine may not effectively prevent HFMD. Secondly, the current EV71 vaccine immunization program excludes infants aged 0–6 months, resulting in an immunization gap within this population [30]. This vaccine is not included in the Expanded Programme on Immunization (EPI) [15]. We have observed variations in reported vaccination rates across different provinces in China. These rates range from as low as 5.53%–15.01% in regions with lower socioeconomic levels, such as Yunnan [5], to as high as 54.82% in areas with higher socioeconomic levels, such as Fujian [15]. However, these numbers are significantly below the recommended coverage of 80%–90%, indicating insufficient coverage of vaccination to mitigate the HFMD pandemic [31], which may also partially explain the unfavored situation of HFMD after the vaccine implementation time. Therefore, to effectively prevent the risk of HFMD, a reassessment of the vaccine strategy is essential. Primary considerations should include incorporating the HFMD vaccine into the EPI and focusing on developing a multivalent vaccine targeting the predominant viral strains. Additionally, exploring the expansion of vaccine coverage to infants below 6 months of age is crucial for reducing the infection risk in this susceptible age group.

Our analysis, stratified by place of residence, revealed significant differences in the risk of HFMD. Following the implementation of the vaccine policy, the relative increase in HFMD risk was more pronounced in Han Chinese areas compared to minority areas, particularly under the extreme Discomfort Index conditions. The risk of HFMD varies between Han Chinese and minority areas, correlating with their distinct cultural practices, customs, behaviors, hygiene habits, and childcare methods. In Han Chinese areas, the higher incidence rate may be attributed to these regional differences. Furthermore, the implementation of vaccination policies has led to a reduction in infections caused by the EV71 virus [15], while cases caused by Cox A16, or other enteroviruses have significantly increased (refer to S5 Fig). This indicates that the implementation of vaccination policies affects the epidemic patterns of HFMD and results in a shift in the predominant viral subtypes. Additionally, the differential sensitivity of various viral subtypes to temperature and humidity influences the transmission and infection of HFMD viruses [32], potentially further explaining the regional differences between Han Chinese and minority areas. Due to variations in host susceptibility to different virus strains, individuals in different populations exhibit varying levels of susceptibility [33], influencing the risk of contracting HFMD. Therefore, the formation of these regional differences is a complex outcome resulting from the interplay of various factors, including population characteristics, the implementation of vaccination policies, and environmental factors. Future research should delve into ethnic-related factors contributing to these differences.

Analysis by sex showed that susceptibility to HFMD changed between the two periods, with boys at higher risk of HFMD after the implementation of the vaccine policy. This aligns with existing literature on sex susceptibility differences in HFMD [34]. These results suggest that there has been a shift in susceptibility among the population following the implementation of the vaccine policy. Research has confirmed the presence of sex differences in child-rearing practices, with boys being encouraged to exhibit more outgoing behavior and engage in outdoor physical activities, while girls are encouraged to adopt quieter behaviors [35]. These differences in parenting approaches may result in boys having more exposure to HFMD pathogens, thereby increasing their susceptibility to infection. Therefore, it is important for parents to pay attention to the health awareness and hygiene habits of boys [36]. This can effectively reduce the transmission routes of HFMD and mitigate the risk of infection. In addition, the differences in HFMD risk between the two economic zones observed in the stratified analyses mainly stemmed from socioeconomic factors such as GDP per capita and population density [37,38]. This study suggests that the necessity for future research to thoroughly consider factors such as race, sex, and economic level when assessing the intervention effects of vaccine policies.

This study has notable strengths, including a large and comprehensive sample derived from long-term county-level time series data, allowing for robust analysis. It examines the combined effects of temperature and relative humidity on HFMD, as well as assesses the impact of EV71 vaccine policy implementation on this basis. This study also provides valuable insights into the interplay between meteorological factors, vaccination strategies, and the risk of HFMD occurrence. Furthermore, expanding the study area to include western China enhances our understanding of the HFMD patterns across diverse geographical regions. However, we must recognize some potential limitations of the study. First, our study falls within the realm of ecological analysis and may be subject to the inherent biases associated with ecological studies. To address this issue, we chose to analyze at the county level to provide a more refined assessment. Second, there are some limitations in terms of delving deeper into and validating the underlying pathological mechanisms because the study data were derived from monitoring data. Third, as in other recent studies [37,38], we chose to exclude data during the COVID-19 pandemic period to ensure the reliability of our findings, considering that the pandemic may lead to changes in healthcare resources, public health policies, and public behavior which could affect the incidence of HFMD [39,40]. In addition, despite our efforts to consider a variety of factors, the presence of unknown confounding variables may affect our interpretation of the effects of vaccine policies.

## Conclusion

In conclusion, our study revealed a nonlinear association between HFMD incidence and the Discomfort Index. Although the EV71 vaccination policy has been implemented, it is noteworthy that the overall risk of HFMD remains inadequately controlled. Due to the influence of vaccination policy, the predominant viral subtypes of HFMD may undergo changes, thereby altering the susceptibility of the population. This highlights the complexity of disease dynamics and the need for an integrated approach in public health strategies. In addition, our research underscores the moderating effects of vaccine intervention may also be influenced by factors such as race, sex, and economic level. This finding emphasizes that these factors should be key considerations in the development of effective vaccine policies.

## Supporting information

S1 Fig The incidence of HFMD in Guizhou Province, China, during the summer months (May to September) from 2012 to 2019. (The dotted line divides the study period into two phases.)
(TIF)

S2 Fig Lag-response relationships between the Discomfort Index and HFMD at the 75th, 95th, and 99th percentiles of the Discomfort Index distribution. (TIF)

S3 Fig Lag-response associations at the Discomfort Index exposure of different subgroups for 2012–2016 and 2017–2019 (with 95% CI). These curves are computed for the temperature corresponding to the 99th percentile.
(TIF)

S4 Fig Comparison of exposure-response and lag-response relationship curves for the Discomfort Index and Humidex as exposure factors for HFMD in Guizhou Province, China (2012–2019). (TIF)

S5 Fig Composition ratio of HFMD virus subtypes in Guizhou Province, 2012–2019. (TIF)

S1 Table The choice of degrees of freedom for the exposure-response function and the lag-response function. (DOCX)

S2 Table The choice of degrees of freedom for air pressure and precipitation. (DOCX)

S3 Table Descriptive statistics of HFMD cases among people ≤5 years of age in Guizhou, China, during the summer months of 2012–2019. (DOCX)

S4 Table Temporal changes of the *RR* of HFMD in the total population and subgroups (among people ≤5 years of age) in different periods (total, 2012–2016, and 2017–2019). (DOCX)
