# Supplementary figures and images for: Assessing the modification impact of vaccination on the relationship of the Discomfort Index with hand, foot, and mouth disease in Guizhou: A multicounty study
Source: PLoS Negl Trop Dis. 2024 Jul 1;18(7):e0012008. doi: 10.1371/journal.pntd.0012008 (PMC11216560; doi:10.1371/journal.pntd.0012008)

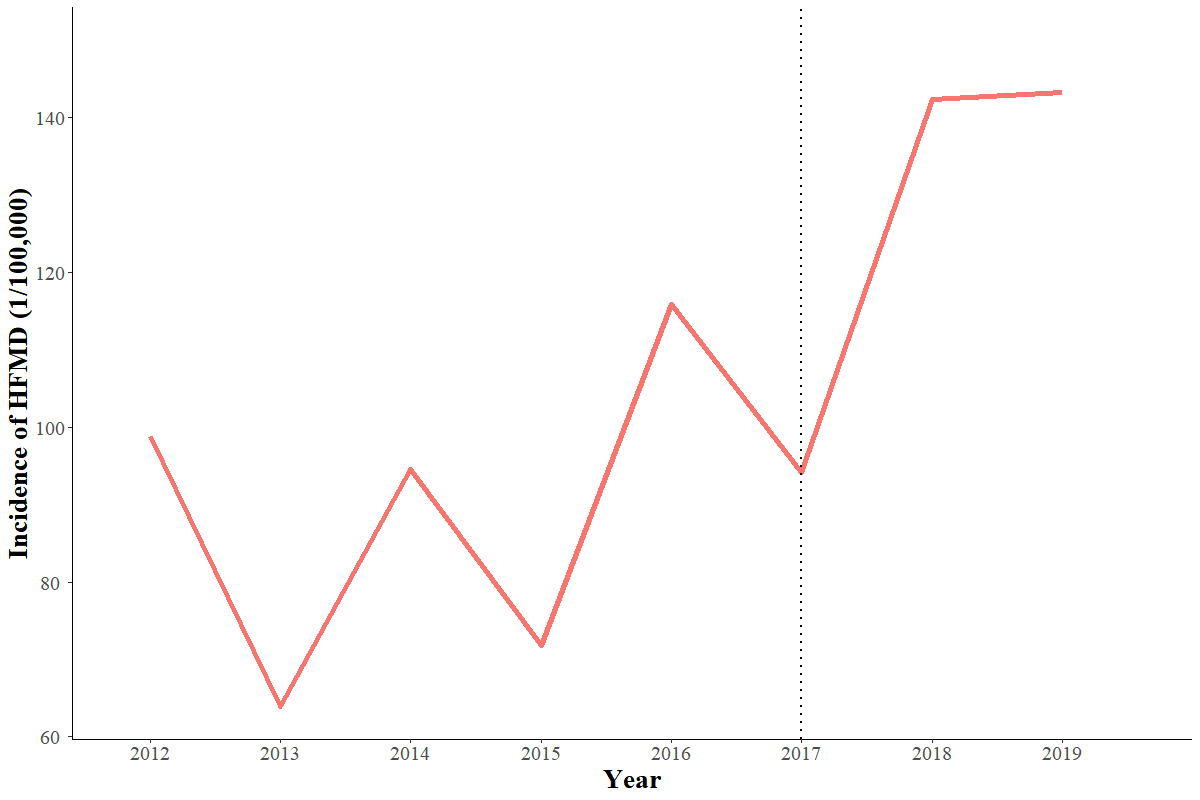

Supplement: S1 Fig — (The dotted line divides the study period into two phases.) (TIF) [file pntd.0012008.s001.tif]

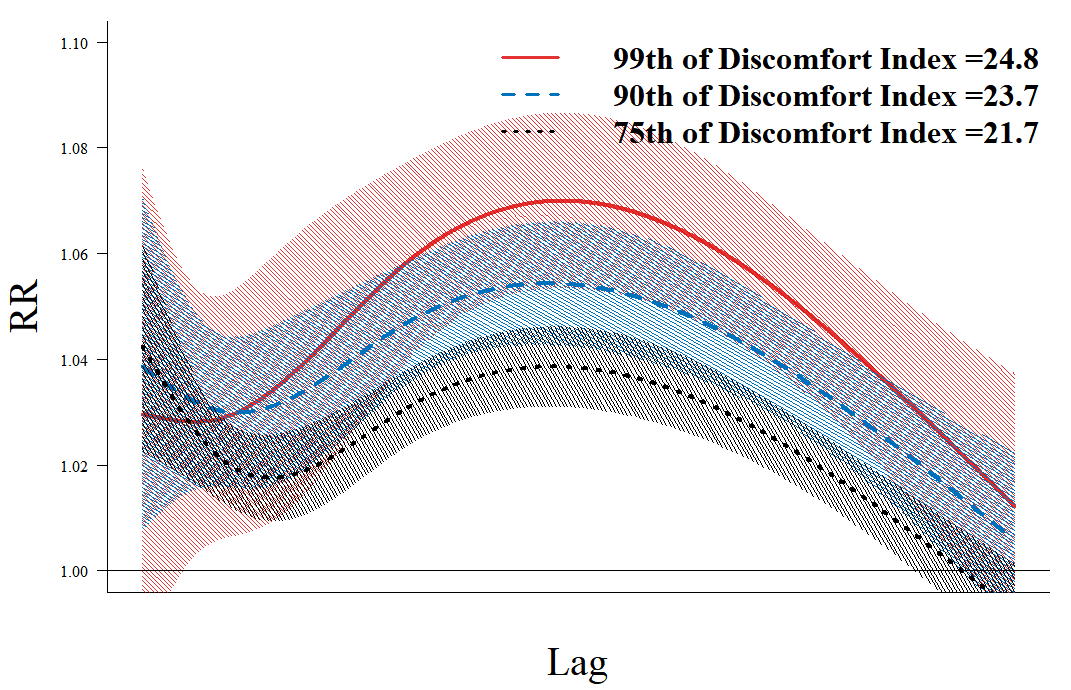

Supplement: S2 Fig — (TIF) [file pntd.0012008.s002.tif]

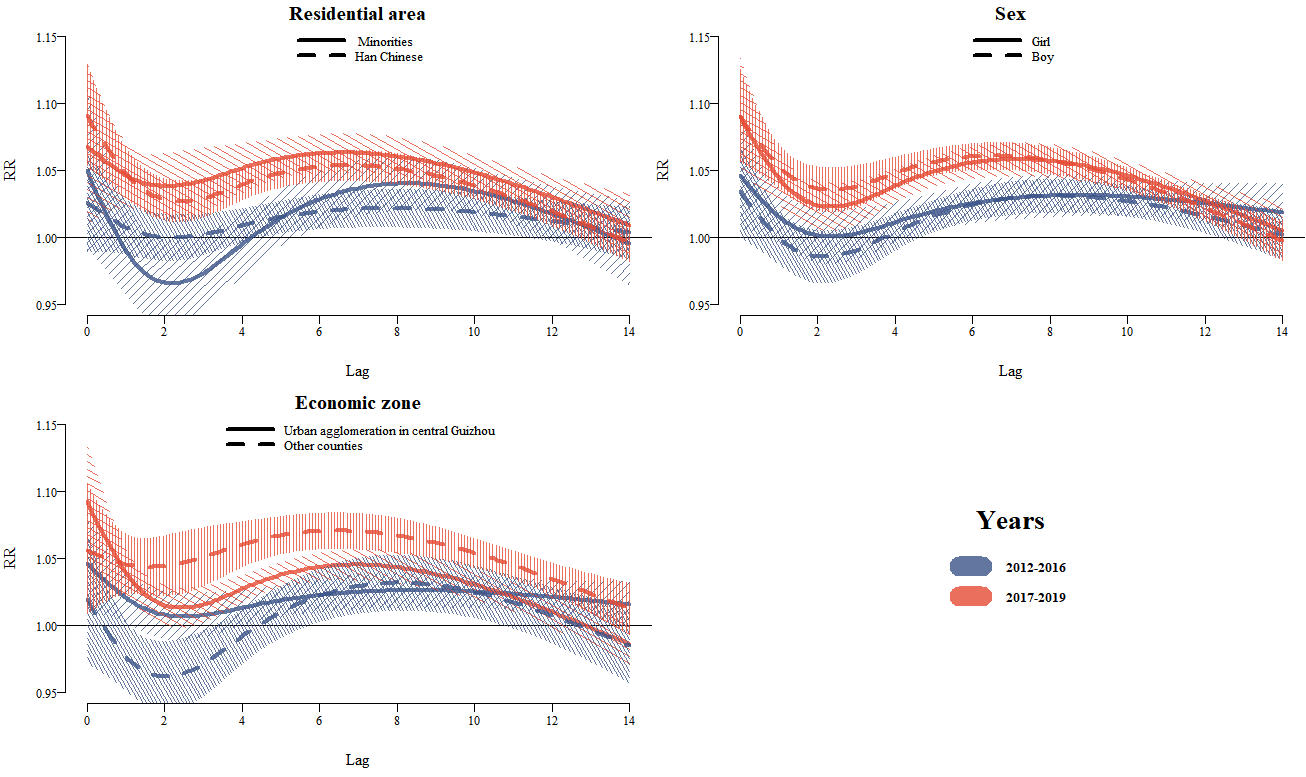

Supplement: S3 Fig — These curves are computed for the temperature corresponding to the 99th percentile. (TIF) [file pntd.0012008.s003.tif]

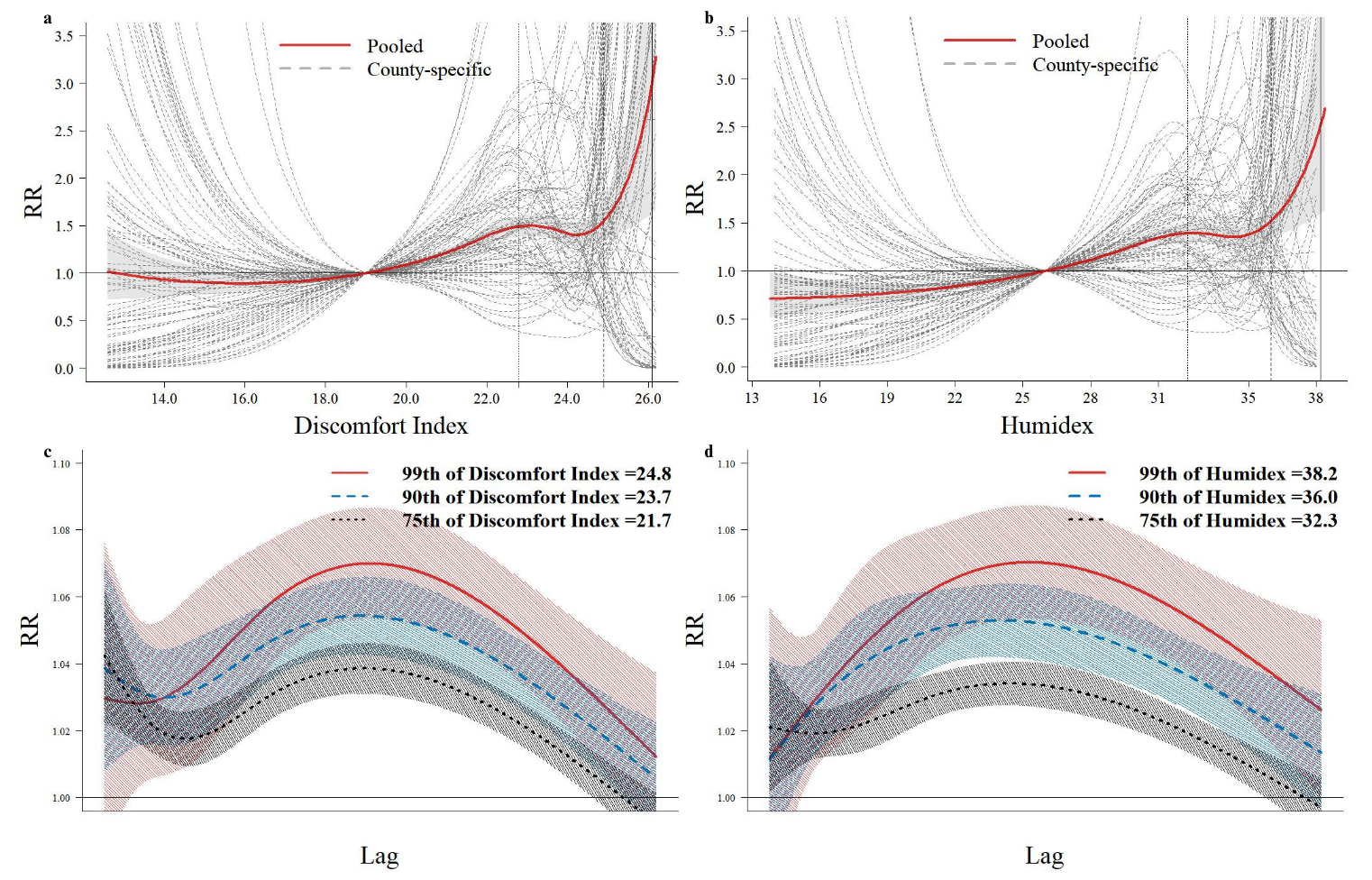

Supplement: S4 Fig — (TIF) [file pntd.0012008.s004.tif]

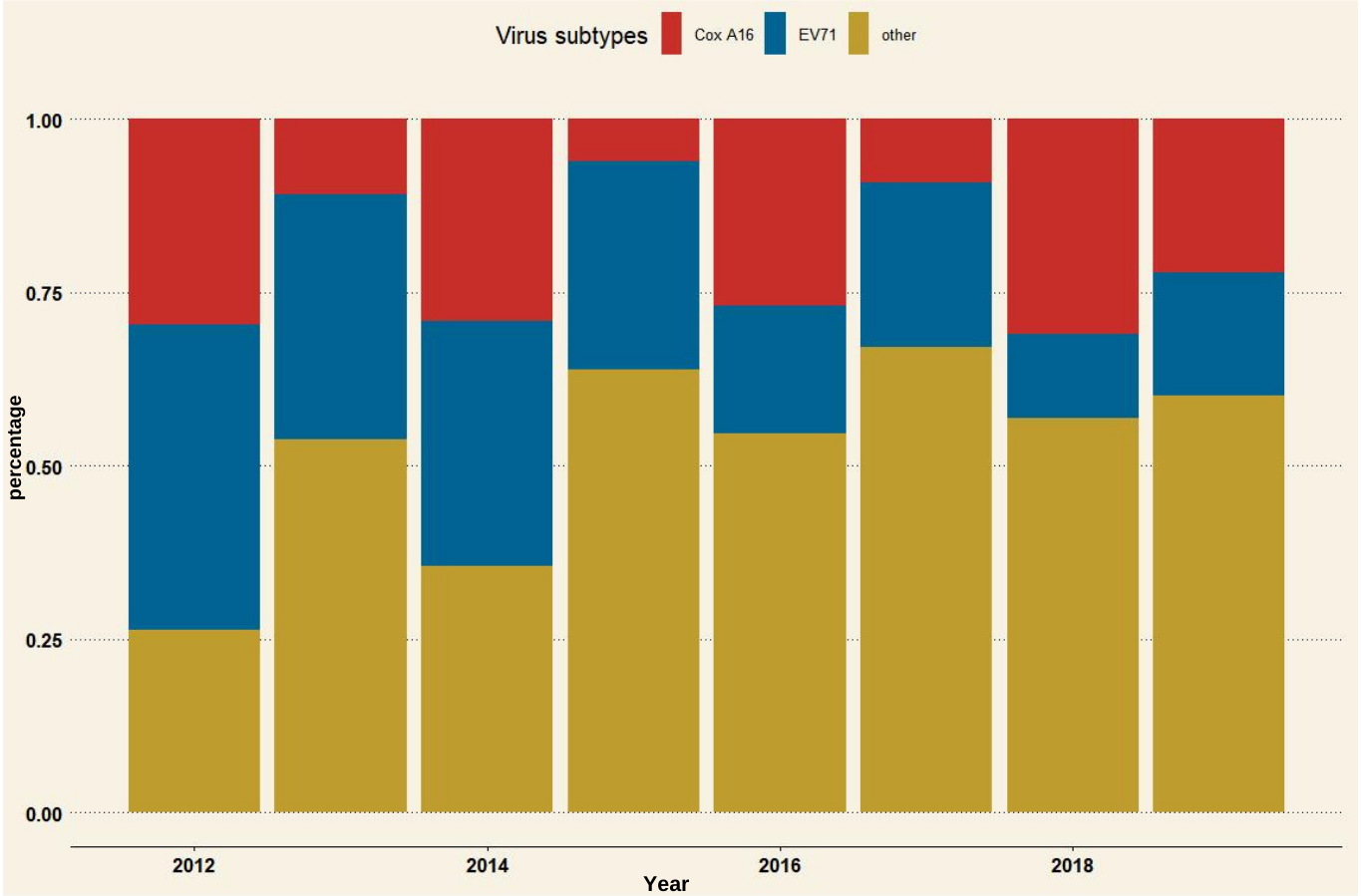

Supplement: S5 Fig — (TIF) [file pntd.0012008.s005.tif]
