# Supplementary material for: Assessing the modification impact of vaccination on the relationship of the Discomfort Index with hand, foot, and mouth disease in Guizhou: A multicounty study
Source: PLoS Negl Trop Dis. 2024 Jul 1;18(7):e0012008. doi: 10.1371/journal.pntd.0012008 (PMC11216560; doi:10.1371/journal.pntd.0012008)
Supplement: S1 Table — (DOCX) [file pntd.0012008.s006.docx]

**S1 Table. The choice of degrees of freedom for the exposure-response function and the lag-response function.**

| **df of the exposure-response function** | **df of the lag-response function** | **Q-AIC** |
| --- | --- | --- |
| 4 | 4 | 3169.4 |
| 4 | 5 | 3172.5 |
| 4 | 6 | 3176.9 |
| 5 | 4 | 3171.8 |
| 5 | 5 | 3176.4 |
| 5 | 6 | 3181.3 |
| 6 | 4 | 3173.3 |
| 6 | 5 | 3179.1 |
| 6 | 6 | 3185.6 |
