# Supplementary material for: Assessing the modification impact of vaccination on the relationship of the Discomfort Index with hand, foot, and mouth disease in Guizhou: A multicounty study
Source: PLoS Negl Trop Dis. 2024 Jul 1;18(7):e0012008. doi: 10.1371/journal.pntd.0012008 (PMC11216560; doi:10.1371/journal.pntd.0012008)
Supplement: S2 Table — (DOCX) [file pntd.0012008.s007.docx]

**S2 Table. The choice of degrees of freedom for air pressure and precipitation.**

| **Air pressure** | **Precipitation** | **Q-AIC** |
| --- | --- | --- |
| 3 | 3 | 3169.4 |
| 3 | 4 | 3170.6 |
| 3 | 5 | 3171.4 |
| 4 | 3 | 3169.4 |
| 4 | 4 | 3170.6 |
| 4 | 5 | 3171.4 |
| 5 | 3 | 3170.3 |
| 5 | 4 | 3171.6 |
| 5 | 5 | 3172.3 |
