# Supplementary material for: Assessing the modification impact of vaccination on the relationship of the Discomfort Index with hand, foot, and mouth disease in Guizhou: A multicounty study
Source: PLoS Negl Trop Dis. 2024 Jul 1;18(7):e0012008. doi: 10.1371/journal.pntd.0012008 (PMC11216560; doi:10.1371/journal.pntd.0012008)
Supplement: S3 Table — (DOCX) [file pntd.0012008.s008.docx]

**S3 Table. Descriptive statistics of HFMD cases among people ≤5 years of age in Guizhou, China, during the summer months of 2012–2019.**

| **Stratification** | **Counties**  **(n)** | **Total cases**  **(n)** | | **Period**  **(year)** | **The Discomfort Index distribution** | | | | |
| --- | --- | --- | --- | --- | --- | --- | --- | --- | --- |
|  |  |  |  |  | **Minimum** | **25th** | **50th** | **75th** | **Maximum** |
| **Total** | 88 | 240863 | 2012–2019 | | 12.563 | 20.011 | 22.085 | 23.638 | 26.264 |
|  |  | 158479 | 2012–2016 | | 12.798 | 19.953 | 21.909 | 23.567 | 26.211 |
|  |  | 82384 | 2017–2019 | | 13.208 | 20.143 | 22.390 | 23.727 | 25.994 |
| **Residential area** |  |  |  | |  |  |  |  |  |
| **Han Chinese areas** | 42 | 192048 | 2012–2016 | | 12.426 | 19.245 | 21.270 | 23.091 | 25.804 |
|  |  |  | 2017–2019 | | 12.392 | 19.434 | 21.763 | 23.248 | 25.563 |
| **Minority areas** | 46 | 48815 | 2012–2016 | | 13.137 | 20.600 | 22.493 | 24.003 | 26.583 |
|  |  |  | 2017–2019 | | 13.953 | 20.791 | 22.962 | 24.164 | 26.388 |
| **Sex** |  |  |  | |  |  |  |  |  |
| **Girl** | 88 | 95439 | 2012–2016 | | 12.798 | 19.953 | 21.909 | 23.567 | 26.211 |
|  |  |  | 2017–2019 | | 13.208 | 20.143 | 22.390 | 23.727 | 25.994 |
| **Boy** | 88 | 145424 | 2012–2016 | | 12.798 | 19.953 | 21.909 | 23.567 | 26.211 |
|  |  |  | 2017–2019 | | 13.208 | 20.143 | 22.390 | 23.727 | 25.994 |
| **Economic zone** |  |  |  | |  |  |  |  |  |
| **Urban agglomeration in central Guizhou** | 33 | 165775 | 2012–2016 | | 12.317 | 19.236 | 21.238 | 22.950 | 25.676 |
|  |  |  | 2017–2019 | | 12.467 | 19.514 | 21.751 | 23.104 | 25.407 |
| **Other counties** | 55 | 75088 | 2012–2016 | | 13.087 | 20.384 | 22.312 | 23.938 | 26.532 |
|  |  |  | 2017–2019 | | 13.653 | 20.521 | 22.773 | 24.100 | 26.347 |
