# Supplementary material for: Assessing the modification impact of vaccination on the relationship of the Discomfort Index with hand, foot, and mouth disease in Guizhou: A multicounty study
Source: PLoS Negl Trop Dis. 2024 Jul 1;18(7):e0012008. doi: 10.1371/journal.pntd.0012008 (PMC11216560; doi:10.1371/journal.pntd.0012008)
Supplement: S4 Table — (DOCX) [file pntd.0012008.s009.docx]

**S4 Table. Temporal changes of the *RR* of HFMD in the total population and subgroups (among people ≤5 years of age) in different periods (total, 2012–2016, and 2017–2019).**

| **Stratification** | **the minimum incidence percentile** | **Period**  **(year)** | **75th *RR*** | **90th *RR*** | **99th *RR*** | ***P-*Value**^a^ |
| --- | --- | --- | --- | --- | --- | --- |
| **Total** | 10th | 2012–2019 | 1.638 (1.463, 1.834) | 1.606 (1.394, 1.851) | 1.622 (1.355, 1.940) |  |
|  |  | 2012–2016 | 1.321 (1.099, 1.588) | 1.230 (1.011, 1.496) | 1.160 (0.929, 1.448) | <0.001 |
|  |  | 2017–2019 | 2.037 (1.796, 2.311) | 2.085 (1.800, 2.414) | 2.442 (1.898, 3.142) |  |
| **Residential area** |  |  |  |  |  |  |
| **Han Chinese areas** | 10th | 2012–2016 | 1.370 (1.133, 1.658) | 1.171 (0.936, 1.465) | 1.154 (0.926, 1.438) | <0.001 |
|  |  | 2017–2019 | 1.699 (1.482, 1.948) | 1.630 (1.404, 1.893) | 2.862 (2.044, 4.007) |  |
| **Minority areas** | 10th | 2012–2016 | 1.483 (1.112, 1.978) | 1.298 (0.949, 1.774) | 1.668 (0.967, 2.874) | 0.021 |
|  |  | 2017–2019 | 1.781 (1.431, 2.216) | 2.038 (1.622, 2.560) | 2.274 (1.448, 3.571) |  |
| **Sex** |  |  |  |  |  |  |
| **Girl** | 10th | 2012–2016 | 1.394 (1.145, 1.697) | 1.246 (0.978, 1.588) | 1.143 (0.872, 1.497) | 0.002 |
|  |  | 2017–2019 | 1.865 (1.605, 2.167) | 2.007 (1.634, 2.465) | 1.660 (1.036, 2.659) |  |
| **Boy** | 11th | 2012–2016 | 1.421 (1.205, 1.675) | 1.188 (0.993, 1.421) | 1.186 (0.942, 1.493) | <0.001 |
|  |  | 2017–2019 | 1.804 (1.559, 2.086) | 1.796 (1.563, 2.064) | 3.214 (2.444, 4.227) |  |
| **Economic zone** |  |  |  |  |  |  |
| **Urban agglomeration in central Guizhou** | 10th | 2012–2016 | 1.587 (1.350,1.866) | 1.381 (1.153, 1.655) | 1.275 (0.983, 1.654) | <0.001 |
|  |  | 2017–2019 | 1.466 (1.283, 1.675) | 1.484 (1.278, 1.724) | 2.356 (1.686, 3.291) |  |
| **Other counties** | 10th | 2012–2016 | 1.243 (0.946, 1.632) | 1.041 (0.763, 1.419) | 1.297 (0.892, 1.888) | 0.004 |
|  |  | 2017–2019 | 2.028 (1.684, 2.442) | 2.026 (1.635, 2.510) | 2.678 (1.703, 4.210) |  |
|  |  |  |  |  |  |  |

^a^ A multivariate Wald test with interaction terms was used to assess the significance of changes before and after the implementation of the EV71 vaccine policy. The null hypothesis is that there is no difference in *RR* before and after the implementation of the EV71 vaccine policy.
